# Impaired regeneration in calpain-3 null muscle is associated with perturbations in mTORC1 signaling and defective mitochondrial biogenesis

**DOI:** 10.1186/s13395-017-0146-6

**Published:** 2017-12-14

**Authors:** Mehmet E. Yalvac, Jakkrit Amornvit, Cilwyn Braganza, Lei Chen, Syed-Rehan A. Hussain, Kimberly M. Shontz, Chrystal L. Montgomery, Kevin M. Flanigan, Sarah Lewis, Zarife Sahenk

**Affiliations:** 1 Center for Gene Therapy, The Research Institute at Nationwide Children’s Hospital, Columbus, OH USA; 2 Department of Pediatrics and Neurology, Nationwide Children’s Hospital and The Ohio State University, Columbus, USA; 3 Department of Pathology and Laboratory Medicine, Nationwide Children’s Hospital, Columbus, OH USA; 4 Neuromuscular Pathology, Nationwide Children’s Hospital, 700 Children’s Drive Rm WA 3024, Columbus, USA; 5 Current Address: King Chulalongkorn Memorial Hospital and Department of Medicine, Faculty of Medicine, Chulalongkorn University, Bangkok, Thailand

**Keywords:** LGMD2A, Skeletal muscle regeneration, Impaired radial growth, mTORC1, Mitochondrial biogenesis

## Abstract

**Background:**

Previous studies in patients with limb-girdle muscular dystrophy type 2A (LGMD2A) have suggested that calpain-3 (CAPN3) mutations result in aberrant regeneration in muscle.

**Methods:**

To gain insight into pathogenesis of aberrant muscle regeneration in LGMD2A, we used a paradigm of cardiotoxin (CTX)-induced cycles of muscle necrosis and regeneration in the CAPN3-KO mice to simulate the early features of the dystrophic process in LGMD2A. The temporal evolution of the regeneration process was followed by assessing the oxidative state, size, and the number of metabolic fiber types at 4 and 12 weeks after last CTX injection. Muscles isolated at these time points were further investigated for the key regulators of the pathways involved in various cellular processes such as protein synthesis, cellular energy status, metabolism, and cell stress to include Akt/mTORC1 signaling, mitochondrial biogenesis, and AMPK signaling. TGF-β and microRNA (miR-1, miR-206, miR-133a) regulation were also assessed. Additional studies included in vitro assays for quantifying fusion index of myoblasts from CAPN3-KO mice and development of an in vivo gene therapy paradigm for restoration of impaired regeneration using the adeno-associated virus vector carrying *CAPN3* gene in the muscle.

**Results:**

At 4 and 12 weeks after last CTX injection, we found impaired regeneration in CAPN3-KO muscle characterized by excessive numbers of small lobulated fibers belonging to oxidative metabolic type (slow twitch) and increased connective tissue. TGF-β transcription levels in the regenerating CAPN3-KO muscles were significantly increased along with microRNA dysregulation compared to wild type (WT), and the attenuated radial growth of muscle fibers was accompanied by perturbed Akt/mTORC1 signaling, uncoupled from protein synthesis, through activation of AMPK pathway, thought to be triggered by energy shortage in the CAPN3-KO muscle. This was associated with failure to increase mitochondria content, PGC-1α, and ATP5D transcripts in the regenerating CAPN3-KO muscles compared to WT. In vitro studies showed defective myotube fusion in CAPN3-KO myoblast cultures. Replacement of CAPN3 by gene therapy in vivo increased the fiber size and decreased the number of small oxidative fibers.

**Conclusion:**

Our findings provide insights into understanding of the impaired radial growth phase of regeneration in calpainopathy.

**Electronic supplementary material:**

The online version of this article (10.1186/s13395-017-0146-6) contains supplementary material, which is available to authorized users.

## Background

Limb-girdle muscular dystrophy, LGMD, type 2A (calpainopathy) is one of the most common forms of LGMD worldwide and is caused by mutations in the *CAPN3* gene, which encodes a skeletal muscle-specific Ca^2+^-activated nonlysosomal cysteine protease, CAPN3 (CAPN3) [1]. CAPN3 is involved in the cleavage and/or breakdown of multiple key skeletal muscle proteins, in particular those involved in the assembly and scaffolding of myofibrillar proteins such as titin, filamin C, vinculin, C-terminal binding protein 1 and others [2–4]. The loss of this activity, which is presumably involved in sarcomere maintenance and turnover, has been implicated in the pathogenesis of LGMD2A [2, 5–7]. In addition, CAPN3 possesses thiol-dependent proteolytic activity specifically directed against the skeletal muscle ryanodine receptor (RyR), a Ca^2+^-release channel [8]. It has been proposed that the dysregulation of skeletal muscle functions in LGMD2A is, at least in part, a consequence of the lack of RyR regulation by CAPN3 [9–11].

In a previous study, we examined the histopathological stages, Pax7-positive satellite cell (SC) content, and muscle-specific microRNA expression in biopsy specimens from well-characterized LGMD2A patients to gain insight into disease pathogenesis. We identified three distinct stages of pathological changes that represented the continuum of the dystrophic process from prominent inflammation with necrosis and regeneration to prominent fibrosis, correlating with age and disease duration [12]. Pax7-positive SCs were highest in the fibrotic group and correlated with microRNA dysregulation as downregulation of miR-1, miR-133a, and miR-206. These observations strongly indicated that miR-206 and miR-1 participate in a regulatory manner that allows transition of SCs from proliferation to differentiation and that the absence or attenuation of this transition results in an excessive number of Pax7-positive SCs, impaired myofiber repair/regeneration, and consequent increased fibrosis. Another underappreciated but important clue to impaired regeneration is the marked overrepresentation of small- and medium-size lobulated fibers expressing type 1 fiber histochemical markers in the LGMD2A biopsies from patients with a long clinical course [12–14].

In the present study, we used a paradigm of cardiotoxin (CTX)-induced cycles of muscle necrosis and regeneration to simulate the early features of LGMD2A in the CAPN3 null (CAPN3-KO) mice and reproduced excessive numbers of small lobulated fibers belonging to slow twitch oxidative (STO) metabolic phenotype as the hallmark finding of impaired/attenuated regeneration. The regeneration paradigm we used here is a versatile model enabling us to assess not only the temporal evolution of this process histopathologically, but also to study the underlying molecular changes in tissue samples in which widespread and synchronous regenerative process is taking place. This is especially important for the CAPN3-KO model with meager histopathological changes in the muscle unless it is switched into the regeneration mode, a high energy requiring state [15, 16]. We pursued a global approach assessing the key regulators of the pathways involved in various cellular processes, components of cell metabolism involved in protein synthesis, energy status, and cell stress. We found that the regeneration in CAPN3-KO muscle was accompanied by a perturbed Akt/mTORC1 signaling pathway, uncoupled from protein synthesis. In addition, we found highly phosphorylated AMP-activated protein kinase (AMPK), the main sensor for ATP shortage in the cell and AMPK-induced growth arrest through inhibition of mTORC1. At 4 weeks post CTX injection, these findings were associated with a failure to increase mtDNA content, mitochondria biogenesis, and ATP synthase transcripts in the CAPN3 null muscles indicating a significant energy and protein synthesis deficit compared to the WT response. Moreover, we found significantly increased transforming growth factor β (TGF-β) transcription levels in the regenerating CAPN3-KO muscles and downregulation of myomiRs correlating with increased connective tissue content associated with impaired radial growth in CAPN3-KO muscle. In addition, our in vitro studies showed defective myotube fusion in CAPN3-KO myoblast cultures suggesting that lobulated fibers might be the consequence of attenuated/incomplete fusion of myotubes. Furthermore, we corrected this aberrant regeneration process in the CAPN3 null muscles in an in vivo study by replacing the missing protein via intramuscular injection of an adeno-associated virus (AAV) vector carrying the *CAPN3* gene.

Our findings may have implications for other dystrophies and chronic acquired myopathic conditions in which attenuated regeneration/impaired radial growth following segmental necrosis and associated fibrosis are the histopathological features.

## Methods

### Cardiotoxin injections for multiple cycles of necrosis and regeneration

Mice were anesthetized with inhaled isoflurane and injected with CTX (Sigma, #C9759, USA) (diluted to 10 μM with sterile saline) every week for a total of six rounds. Thirty and 50 μl of CTX was injected into the left tibialis anterior (TA) and left gastrocnemius muscles, respectively, of 4-month-old CAPN3-KO mice and aged-matched controls. Groups of mice were euthanized, and their muscles harvested at 4 weeks (6.5 months old) and 12 weeks (8.5 months old) post final injection of cardiotoxin (*n* = 3 to 5 mice per strain per time point) and processed for cryostat sectioning. Sections from the same tissue blocks from these injected (left) and uninjected (right) gastrocnemius muscles were utilized for RNA isolation for qPCR and protein isolation for western blots.

### Morphometric studies

#### Muscle fiber diameter distribution

Succinic dehydrogenase (SDH) enzyme histochemistry was used to assess metabolic fiber-type differentiation [slow twitch oxidative (STO), fast twitch oxidative (FTO), and fast twitch glycolytic (FTG)] using standard protocol established in our laboratory. Muscle fiber type-specific diameter measurements were obtained using 12-μm-thick SDH-stained cross sections at 4 and 12 weeks after final cardiotoxin injection. Three images, each representing three distinct zones of the gastrocnemius muscle (a deep zone predominantly composed of STO, intermediate zone showing a checkerboard appearance of STO and FTO or FTG, and the superficial zone predominantly composed of FTG fibers) along the midline axis (per section per animal) were photographed at ×20 magnification using an Olympus BX41 microscope and SPOT camera (Olympus BX61, Japan). This approach was chosen to capture the alterations in the oxidative state of fibers in each zone in response to metabolic changes during regeneration. Diameters of dark (STO), intermediate (FTO), and light (FTG) fibers were determined by measuring the shortest distance across the muscle fiber using Zeiss Axiovision LE4 software (v.4.8). The fiber diameter histograms were generated separately for STO; FTG and FTO were combined to represent the total fast twitch fiber population (FTG/O), derived from three animals and expressed as number per square millimeter of endomysial area (mean ± SEM). The mean fiber diameter was derived from combining all three fiber types. An average of 900–1700 fibers were measured per group. TA muscles were used for assessment of fibrosis (see below).

### Dual label IgG immunofluorescence for fiber typing

Twelve 12-μm thick cryosections from gastrocnemius muscles were mounted on glass microscope slides, were allowed to come to room temperature, with no fixation, and outlined in PAP pen. Immune staining was done using an automated stainer (BioGenex i6000 Automated Staining System, San Ramon, CA, USA) which performed the following steps at room temperature. Tissue sections were blocked with 10% goat serum, 0.1% Tween 20-TBS for 1 h, incubated with mixture of two primary antibodies (MHC Type I (BA-D5) and MHC Type IIA (SC-71); Developmental Studies Hybridoma Bank, University of Iowa, Iowa City, IA) diluted 1:50 in TBS for 1 h, followed by three consecutive washes with TBS for 20 min. Sections were then blocked for 30 min, incubated with mixture of two goat anti-mouse IgG secondary antibodies (Alexa Flour 594 Mouse IgG1 for detecting SC-71 and Alex Flour 488 Mouse IgG2b for detecting BA-D5; Life Technologies) diluted 1:200 in TBS, washed three times with TBS for 20 min, and mounted with Vectasheild (Vector Labs, Burlingame, CA). Images were captured using a Zeiss Axioskop 2 microscope (Zeiss, Thornwood, NY).

### Assessment of fibrosis (endomysial and perimysial connective tissue)

#### Method of quantitation

The amount of endomysial and perimysial connective tissue was quantitated in TA muscle using picrosirius red stain using a protocol established in our laboratory for comparison of CAPN3-KO and WT control mice at 4 (*n* = 3) and 12 (*n* = 4) weeks after 6 cycles of necrosis/regeneration [17]. Five images to cover central area and four quadrants of the muscle were photographed at ×20 from each mouse in both groups; the level of fibrosis was analyzed with ImagePro software (v.6.0). Analysis was made using customs method with 2.5-min counter stained slides without color correction. Red area (as proportion of fibrotic area) was expressed as percent of total area. The mean ± SEM of five images represented each mouse.

### rAAV production

DNA fragment sequence including open reading frame of mouse *CAPN3* (NM_007601.3) between two Not1 restriction sites was synthesized (this service is provided by Eurofin Genomics, USA) and subcloned into our single strand AAV.MCK (muscle creatine kinase) vector [18]. After the sequences were confirmed, the vector was given to our Viral Vector Core at Nationwide Children’s Hospital, Columbus, for generation and purification of viral vector with AAV9 serotype. The final titer (vg ml^−1^) was determined by quantitative reverse transcriptase PCR using the specific primers and probes for MCK promoter utilizing a Prism 7500 real-time detector system (PE Applied Biosystems, Grand Island, NY, USA). Aliquoted viruses were kept in − 80 °C until use.

#### In vivo *AAV.CAPN3* gene therapy

To assess if WT CAPN3 restores the impaired regeneration process, TA muscles from CAPN3-KO mice (*n* = 4) under anesthesia were first injected with CTX, 30 μl, and 2 weeks later were transduced to express WT CAPN3 using AAV9.MCK.CAPN3 vector at 1 × 10^11^ vector genomes (vg), in 20-μl volume via intramuscular injection. TA muscles from another cohort of CAPN3-KO (*n* = 4), served as controls, received the same volume of PBS 2 weeks post CTX injection. Mice were killed at 6 weeks post CTX injection; TA muscles were removed and processed for cryostat sectioning. Twelve-micrometer thick cross sections were first stained with H&E for routine histopathological evaluation; muscle fiber type-specific diameter measurements were obtained from SDH-stained cross sections of the TA from three mice in each group as described above. Three random images of the TA (per section per animal) was photographed ×20 magnification and the fiber diameter measurements and fiber type-specific histograms were generated as described above. An average of 700–1000 fibers were measured per group.

### Primary muscle cell culture and myotube formation

Primary myoblasts were derived from the hind limb muscles of 1-month-old CAPN3 KO (*n* = 5) mice and age-matched WT C57BL/6 (n = 5) mice; the protocol was adapted from previous studies [19, 20]. Briefly, hind limb muscles were dissected, minced, and subjected to collagenase (5 mg/ml) digestion for 1 h at 37 ^°^C. The digest was then mechanically dissociated by repeated trituration using 5-ml syringe with 18 G needle followed by filtration through a 100-μm vacuum filter (Millipore, NY, USA). After centrifugation, mono-nucleated cells were resuspended in myoblast growth medium, HAM’S F10 Medium (Gibco-Invitrogen, #11550-043, Carlsbad, CA) supplemented with 20% FBS (Fisher Scientific, #26-140-079 Carlsbad, CA), penicillin/streptomycin (Gibco-Invitrogen, #15640055 Carlsbad, CA)), bFGF (20 ng/ml final concentration, Pepprotech, #450-33, NJ, USA), and pre-incubated on 10-cm cell culture dishes for 1 h. Non-adherent cell was collected, and another 1-h pre-incubation was performed. Non-adherent cells were collected and put into six-well plates coated with matrigel (BD Bioscience, #356234, Franklin Lakes, NJ, USA) incubated until they reach confluence.

For myotube formation assays, the cells were seeded into matrigel-coated six-well plates (2 × 10^5^/well/mouse, *n* = 5) and incubated until they reach confluency for 3 days. Then, myotube formation was induced by changing growth medium to differentiation medium [DMEM (Gibco-Invitrogen, #10569010, Carlsbad, CA) supplemented with 5% horse serum (Gibco-Invitrogen, #26050088, Carlsbad, CA) and 1% penicillin/streptomycin (Invitrogen, #15640055 Carlsbad, CA)]. After 3 days of incubation, the cells were fixed with 4% paraformaldehyde and stained for myosin heavy chain (MHC) (DSHB, #MF-20s, Iowa City, Iowa) to identify the myotube formation. Anti-mouse Alexa 488 secondary antibody (Thermo Fisher, #A11001, Boston, MA, USA) was used to visualize the myotubes with multinuclei stained with DAPI (4′,6-diamidino-2-phenylindole, Sigma, USA). From each well, five randomly selected areas were photographed at ×20 using a fluorescence microscope (Olympus BX61, Japan) and the number of nuclei was determined using ImageJ software. In addition, the lengths of myofibers within each microscopic frame were measured and the number of myonuclei per myotubes was determined. A total of 246 myotubes from CAPN3-KO and 239 from WT cultures were analyzed. Scattergrams were generated by plotting the number of nuclei for each myotubes. In addition, the nuclei density per myotubes was determined and percent distribution histograms (as percent of total myotubes carrying two or more nuclei) were generated from CAPN3-KO and WT specimens for comparison.

### QPCR experiments

Total RNA and DNA were isolated from the left gastrocnemius muscles of CAPN3-KO and WT mice at 4 and 12 weeks following 6 cycles of necrosis and regeneration and from the uninjected right side. For RNA isolation, mirVana miRNA isolation kit (Life Technologies, #AM1560, TX, USA) and for DNA isolation, QIAamp DNA Mini Kit (Qiagen, #51304 CA, USA) were used according to the manufacturer’s protocol. Reverse transcription was performed by using Taqman® microRNA reverse transcription kit (Applied Biosystems, #4366597, Carlsbad, CA) and Transcriptor First Strand cDNA synthesis kit (Roche, # 04379012001 Roche, USA) following manufacturer’s instructions. Predesigned Taqman primers and probes for miR-1, miR-206, miR-133a, and U6snRNA were purchased from Applied Biosystems (Carlsbad, CA). U6snRNA was used as a reference to normalize miRNA qPCR results. Other qPCR experiments were performed by using iTaq™ universal SYBR® Green supermix (Biorad, #1725122, Hercules, CA, USA) using GAPDH as a house keeping gene for the normalization. Primer sequences for TGF-β [21], PGC1α [22], ATP5D [15], GAPDH [23], mtDNA, and nuclear DNA (nDNA) [24] were taken from published materials. The copy numbers mtDNA and nDNA were calculated based on the standard curves made by using DNA from splenocytes of wild type C57BL/6 mouse with the range of 0–100 ng for nDNA versus 0–1 ng for mtDNA. It was assumed that 1 ng DNA includes 150 copies of nDNA and 300,000 copies of mtDNA [25]. The ratio of mtDNA/nDNA was referred as the mitochondria content. All qPCR experiments were done by using ABI 7500 real-time PCR machine and the results were analyzed using Data Assist Software (ABI).

### Protein extraction and western blot experiments

Frozen gastrocnemius muscle blocks were cut in 20-μm thickness and put into an Eppendorf tube (15–20 section per block) and homogenized in lysis buffer [RIPA lysis buffer (Thermo Fisher, #89900, USA) with 1× Halt protease inhibitor (Thermo Fisher, #78429, USA) and 1× phosphatase inhibitor (Sigma, #P0044, USA)] using an automatic pellet mixer and disposable pestles with 20 s periods for three times. The lysates were centrifuged at 13,000 rpm for 10 min at 4 °C, and the supernatants were carefully collected. Protein concentration was measured by using BCA Protein Assay Kit (Thermo Fisher, #23252, Waltham, MA, USA). Protein samples (10–40 μg) were run 4–12% Bolt® Bis-Tris Plus precast 15-well polyacrylamide gels (Thermo Fisher, #NW04120BOX) and transferred to PDVF membranes (GE Healthcare, #10600021, Pittsburgh, USA). Membranes were blocked for 2 h at room temperature with 5% bovine serum albumin (BSA, Bedford, MA, USA) in TBS buffer with 0.05% Tween-20 (TBS-T, Amresco, OH, USA) and incubated with the appropriate primary antibody in TBS-T buffer with 5% BSA overnight in cold room at 4 °C. The primary antibodies used in this study were as follows: anti-phospho S6K1 Thr 389 (#9234), anti-S6K1(#2708), anti-Phospho Akt Thr308 (#2965) anti-Akt (#9272), anti-phospho 4E-BP1 thr37/46 (#2855), anti-4E-BP1 (#9644), anti-phospho AMPKα Thr172 (#2535), anti-AMPK (#2532s) anti-phosphoUlk1 Ser555 (#5869), anti-Ulk1 (#8054 s), anti-phospho Raptor Ser 792 (#2083), anti-Raptor (2280), anti-phospho, LKB1 Ser428 (#C67A3), and anti-LKB (#3047) (Cell Signalling Technologies, Danvers, MA, USA). After washing 5 min for five times on an orbital shaker with TBS-T, the membranes were incubated with secondary antibodies [HRP-conjugated anti-rabbit (#HAF008) IgGs from R&D Systems, Minneapolis, MN, USA] in 5% dry milk in TBS-T for 1 h. The membranes were washed again with TBS-T in the same way as above and then incubated with ECL Prime western detection reagent (Amersham, #RPN2232 NJ, USA) for 1–5 min followed by exposing to X-ray films (Denville, #E3018, MA, USA) using multiple exposure times. Protein bands on the film were pictured using a camera (Sony A600, Japan), and the band intensities were quantified using (Quantity-One software, BioRad, v.4.6.9). The relative content of analyzed phosphorylated proteins in each sample was determined by normalizing band intensities to the non-phosphorylated band intensities in the same sample. The membranes were stained with 0.1% Coomassie Brilliant Blue R stain (Thermo Fisher, USA), rinsed, and photographed to confirm equal protein loading in each lane.

### Statistics

For muscle fiber size comparisons between CTX and saline-injected CAPN3-KO and WT groups, statistical analysis was performed in Graph pad Prism 6 software, using one-way analysis of variance followed by Tukey’s multiple comparison test. Two-tailed *t* test was performed when applicable. Significance level was set at *P* < 0.05. Summary statistics were reported as mean ± SEM.

## Results

### Muscle regeneration is impaired in CAPN3 null muscle

**Fig. 1:**
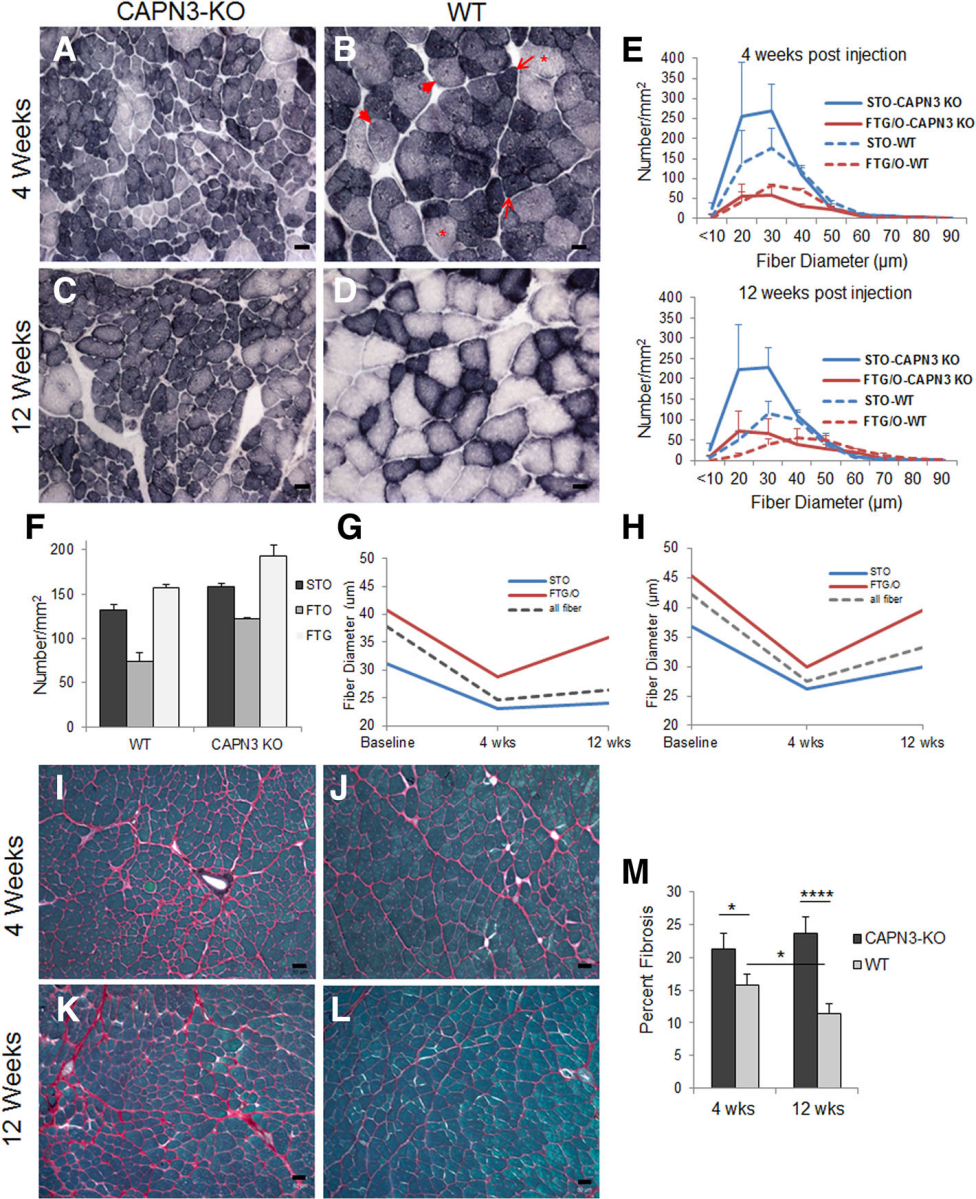
Impaired radial growth of regenerating muscle fibers in CAPN3 null muscle. Regeneration in CTX-induced muscle damage. Representative images of SDH-stained tissue sections of CAPN3-KO (**a**, **c**) and wild type (WT) gastrocnemius muscles (**b**, **d**) at 4 and 12 weeks after last CTX injection, respectively. Slow twitch oxidative (STO, arrows), fast twitch oxidative (FTO, arrow heads) and fast twitch glycolytic (FTG, asterisks) are shown (**b**). In the CAPN3-KO muscle, markedly increased numbers of small STO fibers were present at both time points compared to WT compatible with aberrant regeneration. Scale bar = 30 μm for **a**–**d**. **e** Muscle fiber size distribution histograms (mean ± SEM; derived from three mice in each group) of STO fibers revealed markedly impaired radial growth at 4 weeks (top) and 12 weeks (bottom) after last CTX injection. **f** The fiber type composition of CAPN3-KO and WT from non-regenerating contralateral muscle. At CAPN3-KO baseline, there are more FTO fibers and due to smaller fiber size, the numbers per unite area for all fiber types are more compared to WT. Radial growth dynamics of STO and fast twitch (FTG/O) fibers from CAPN3-KO (**g**) and WT (**h**) within 8 weeks period are shown. Picrosirius red staining for assessment of post regeneration connective tissue in tibialis anterior muscle from CAPN3-KO at 4 (**i**) and 12 weeks (**k**) post CTX injection compared to WT counterparts at 4 (**j**) and 12 weeks (**l**). **m** The percent of connective tissue is significantly higher in the CAPN3-KO than WT at 4 and 12 weeks post injection. The WT muscle showed a significant decrease in the connective tissue content along with increased radial growth process at 12 weeks. Scale bar = 50 μm for **i**–**l**. Error bars represent SEM; *n* = 3 mice per strain per time point. *P < 0.05, ****P < 0.0001 by two-tailed *t* test

In our preliminary experiments, we first established that gastroc muscle from CAPN3-KO mice examined 4 weeks after a single CTX injection resulted in an increase in the number of small size oxidative fibers with a lobulated pattern, while the WT muscle, subjected to the same treatment protocol, showed significantly larger diameter muscle fibers with normal appearing cytoskeleton suggesting impaired radial growth or an attenuated regeneration process in the CAPN3-KO muscle following one cycle of necrosis. In contrast to one-time damage, the muscles from calpainopathy patients with defective or absent CAPN3 activity, particularly in the early stages of the dystrophic process, show an ongoing cycle of necrosis and regeneration [12]. In order to simulate this process, we used CTX-induced multiple cycles of muscle necrosis and regeneration in the gastrocnemius muscles of CAPN3-KO mice and assessed the regeneration process temporally at 4 and 12 weeks after the last CTX injection (Fig. 1a–d). SDH histochemical stain was used to assess the fiber-type differentiation based on their metabolic activity/oxidative state [slow twitch oxidative (STO), fast twitch glycolytic (FTG), and the intermediate fast twitch oxidative (FTO)], along with identification of fibers displaying a lobulated pattern of structural change. In the CAPN3-KO muscle, a marked increase in the numbers of small STO/type 1 fibers was noted at both time points (Table 1; FTG and FTO were combined to represent fast twitch, designated as FTG/O). Using another method, immunohistochemical double staining for type 1/slow myosin heavy chain, MHC (STO), and type 2A (FTO) at 4 and 12 weeks post injury, we confirmed that this increase in the oxidative fibers is indeed predominantly corresponding to type 1 fibers (Additional file 1: Figure S1 A-D). The quantitative studies showed significantly smaller mean muscle fiber diameter in CAPN3-KO compared to WT at both time points (Table 1). This was predominantly due to markedly impaired radial growth of STO fibers, which showed a meager increase in their fiber diameter at 12 weeks compared to 4 weeks after last CTX injection (Table 1, Fig. 1e). Radial growth of fast twitch (FTG/O) fibers appeared attenuated compared to WT, although it was significantly better than STO fibers. Moreover, the ratio of STO:FTG/O was much higher in CAPN3-KO (2.7 at 4 weeks and 4.1 at 12 weeks in CAPN3-KO vs. 1.80 and 1.756 in WT respectively) compared to controls, indicating that in the CAPN3 null muscles following several cycles of necrosis/regeneration, the small fibers with oxidative capacity predominate. It should be noted that the CAPN-3-KO mice have reduced muscle mass, and the baseline fiber diameter of the contralateral (non-regenerating) gastrocnemius muscle was smaller than WT. This size reduction occurred in both fast and slow fibers as previously reported [26] (Table 1). Moreover, the fiber type composition of baseline CAPN3-KO muscle showed more fibers with higher oxidative capacity (STO and FTO) compared to WT (Fig. 1f). The growth dynamics of regenerating WT and CAPN3-KO muscles are shown in Fig. 1g, h. At 12 weeks post injection, the mean fiber diameter of regenerating WT fibers has not reached to baseline values. Both in WT and the mutant muscle, the fast fibers (FTG/O) displayed a similar growth dynamics while the growth rate of STO fibers in the CAPN3-KO remained almost flat from 4 to 12 weeks and the mean fiber diameter stayed parallel to the STO fibers as the consequence of its abundance in number.

**Table 1:** Gastrocnemius muscle fiber size in CAPN3-KO and wild type mice

	Baseline		4 weeks		12 weeks	
	Number per square millimeter	Diameter (μm)	Number per square millimeter	Diameter (μm)	Number per square millimeter	Diameter (μm)
CAPN3-KO						
STO	158	31.16 ± 0.4	647	23.1 ± 0.3****	633	24.1 ± 0.3****
FTG/O	316	40.81 ± 0.4	240	28.8 ± 0.6	153	35.8 ± 0.9**
All fibers	474	37.92 ± 0.3	887	24.6 ± 0.3****	786	26.4 ± 0.3****
Wild Type						
STO	132	36.92 ± 0.5	466	26.2 ± 0.4	321	29.9 ± 0.4
FTG/O	230	45.35 ± 0.4	256	29.9 ± 0.6	183	39.6 ± 0.7
All fibers	362	42.27 ± 0.4	724	27.5 ± 0.3	504	33.3 ± 0.4

^****^ *P* < 0.0001 compared to same wild type parameter

**P < 0.01 compared to same wild type parameter

#### Impaired regeneration in CAPN3 null muscle is associated with fibrosis

Aberrant regeneration resulting from impaired myotube fusion following segmental necrosis was previously proposed as the leading cause of fibrosis [27]. In order to assess fibrosis along with temporal evolution of aberrant regeneration, we used picrosirius red stain for quantifying the endomysial and perimysial connective tissue content in TA muscles from CAPN3-KO and WT mice at 4 and 12 weeks after the last CTX injection (Fig. 1i–l). In CAPN3-KO muscles, the amount of post regeneration connective tissue at 4 and 12 weeks was significantly greater than WT controls (Fig. 1m). The extent of fibrosis at 12 weeks post injection was higher than the earlier time point, although this was not significant, correlating with the attenuated fiber size increase that occurred during this longer post injection recovery period. As expected, the WT muscle showed a significant decrease in the connective tissue content along with an improved radial growth process, i.e., increased fiber diameter. Collectively, these findings recapitulate the hallmark pathological features of LGMD2A, showing an excessive number of small type 1 fibers with lobulated structural appearance and increased endomysial and perimysial connective tissue [12, 13].

### Defective myoblast fusion in CAPN3 null mice and restoration of impaired regeneration with in vivo gene therapy

**Fig. 2:**
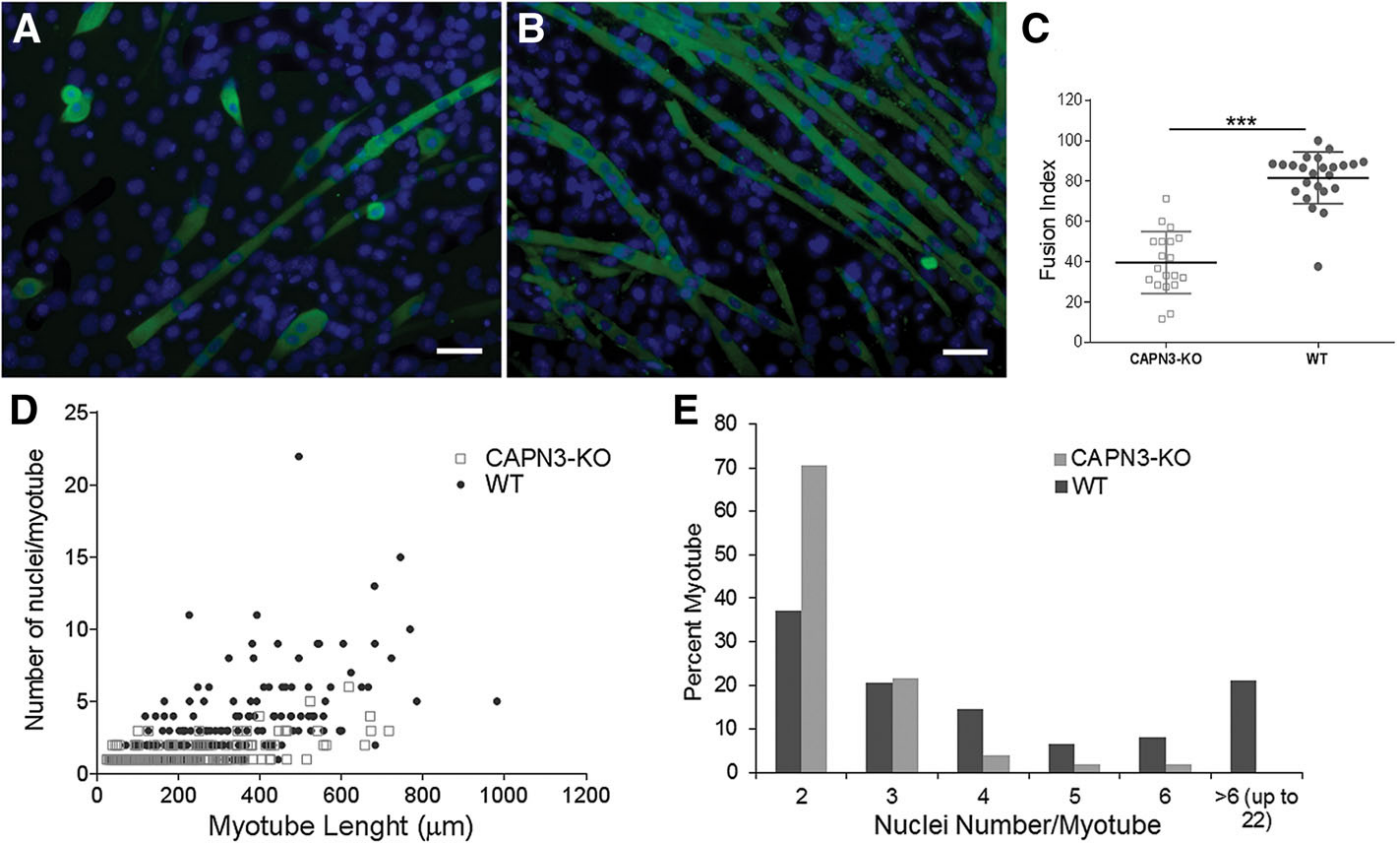
Absence of CAPN3 impairs myoblast fusion. Fluorescent images of primary myoblast cultures isolated from 1-month-old CAPN3-KO (**a**) and WT (**b**) mice differentiated for 3 days on glass coverslips, fixed and stained for MHC with antibody (green) and DAPI for nucleus. Scale bar = 50 μm. **c** Fusion index (mean ± SEM). There was a significant reduction in fusion competence between WT (82%) and CAPN3-KO (40%) myoblasts (*P* < 0.001 by two-tailed *t* test; data derived from five mice per group; 4–5 frame photographed at 20× per mice were analyzed). CAPN3-KO myotubes were shorter and on average had fewer nuclei than wild type myotubes (**d**). A total of 246 myotubes from CAPN3-KO and 239 from BL/6 cultures were analyzed. Scattergrams were generated by plotting the number of nuclei for each myotubes. The nuclei density per myotubes was determined and percent distribution histograms (as percent of total myotubes carrying 2 or more nuclei) were generated for comparison (**e**). ***P < 0.001

Our qualitative and quantitative histopathological studies revealed excessive numbers of small oxidative and lobulated fibers as clues, suggesting that mutant myotubes lacking CAPN3 may be defective in fusing with each other to form strips of multinucleated myotubes therefore failing to complete regeneration process competently. To explore this directly, myoblasts from CAPN3-KO muscles and WT controls were induced to differentiate to form in vitro myotubes. Three days after the induction, the myotubes were stained with an anti-MHC antibody and a nuclear stain to count the number of nuclei that had incorporated into multinucleated myotubes. We observed that overall, the CAPN3-KO myotubes were shorter and a large fraction displayed an ovoid shape with single nucleus or multiple clumped nuclei instead of the elongated shape seen in WT cells (Fig. 2a, b). Only 39.6% of CAPN3-KO cells fused to form multinucleated MHC-positive myotubes, while in WT, 81.7% of cells had fused (Fig. 2c). To quantify this fusion defect further, we measured the length of myotubes and counted the number of nuclei in each myotube. CAPN3-KO myotubes were shorter and on average had fewer nuclei than WT myotubes; 7.8% of multinucleated fibers contained four and more nuclei in CAPN3-KO while in the WT, it was 39.2% (Fig. 2d, e). Taken together, the CTX experiments and myoblast fusion assays demonstrate that both CAPN3-KO mature myocytes and myoblast progenitors are fusion defective. This defect likely contributes to the impaired muscle regeneration and resultant progressive fibrosis as seen in LGMD2A.

#### In vivo gene therapy restores impaired regeneration in CAPN3-KO muscle

**Fig. 3:**
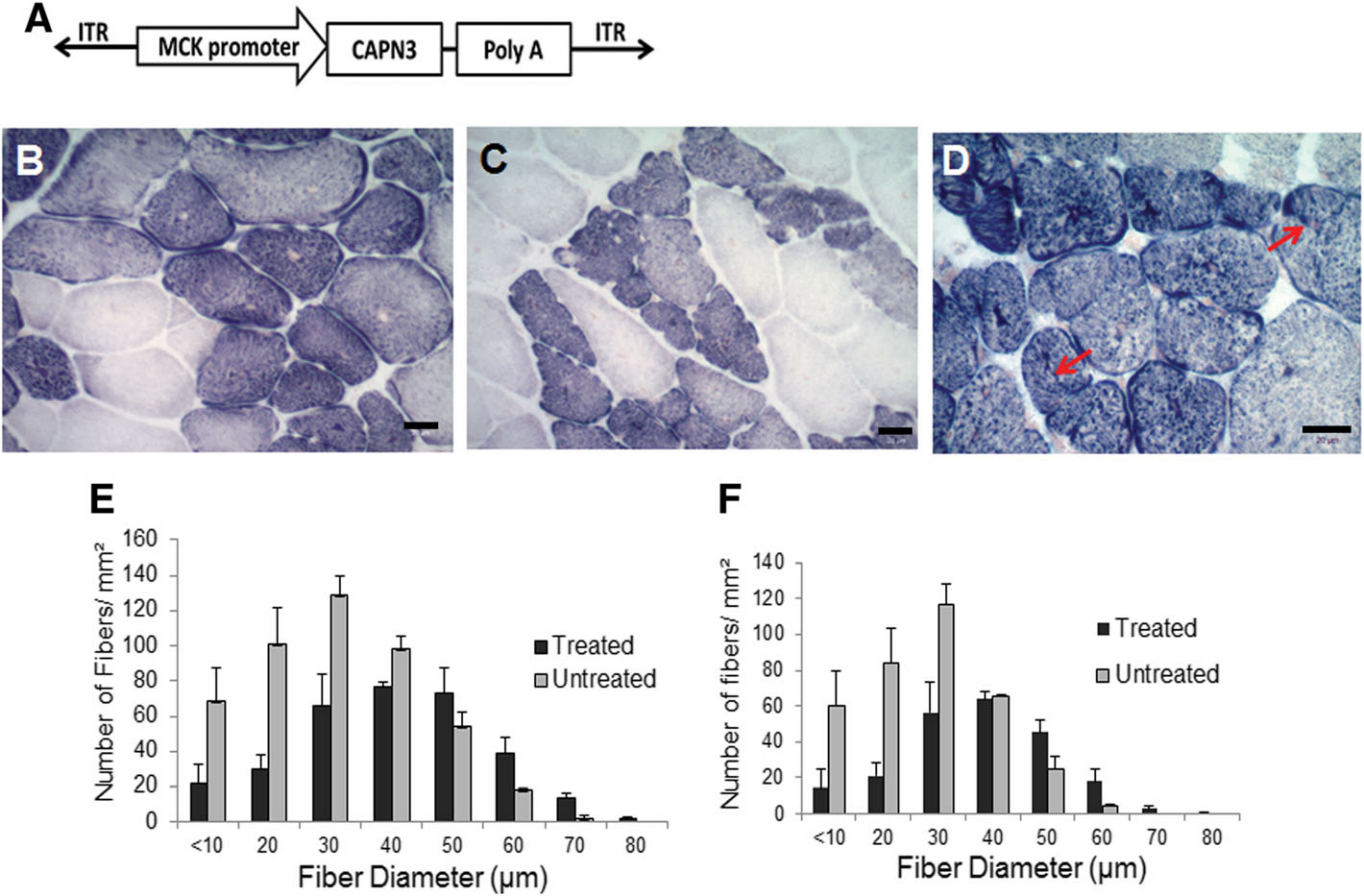
In vivo gene therapy restores impaired regeneration in CAPN3-KO muscle Schematic diagram of single stranded AAV9.CAPN3 vector (**a**). In between the 5′ and 3′ single strand ITRs (inverted terminal repeats), muscle creatine kinase (MCK) promoter (563 bp) drives the expression of CAPN3 open reading frame (2466 bp). Also labeled is polyadenylation site (Poly A, 53 bp). Tibialis anterior (TA) muscles from CAPN3-KO mice were first injected with CTX, and 2 weeks later with 1 × 10^11^ vg of AAV.CAPN3 to left TA (**b**) or PBS to right TA (**c**). Four weeks after gene injection the muscle diameter increased and the lobulated fibers were less common compared to the untreated CAPN3-KO muscle. **d** Lobulated fibers with a pattern of subsarcolemmal organelle, mitochondria distribution (arrows) suggesting partial myotube fusion in the untreated CAPN3-KO muscle at higher magnification. Scale bar = 20 μm for B-D. **e** The muscle fiber size distribution histograms (mean ± SEM/square millimeter area; derived from 3 mice in each group) of the treated and untreated TA muscle from CAPN3-KO mice showing a shift to larger diameter fibers with treatment; an increase in the small diameter subpopulation is present in the untreated group. **f** STO fiber size distribution histograms show excessive number of small fibers in the untreated CAPN3-KO muscle.

To assess if restoring CAPN3 attenuates the impaired regeneration, we designed an AAV vector carrying the CAPN3 gene under MCK muscle-specific promoter (AAV.CAPN3) (Fig. 3a). TA muscles from CAPN3-KO mice under anesthesia were first injected with CTX and 2 weeks later with 1 × 10^11^ vg of AAV.CAPN3. Four weeks after gene injection, a significant increase in muscle diameter with an apparent decrease of internal nuclei and far less number of small fibers with lobulated pattern was observed (Fig. 3b). The untreated CAPN3-KO muscle had 31.6% more fibers per square millimeter area, mostly composed of small and lobulated STO fibers indicating that the treatment improved myotube fusion, therefore decreased individual small fiber number per unit area (Fig. 3c, d; Table 2). The fiber size distribution histograms of the treated TA muscle showed a shift to larger diameter fibers with treatment, and the excessive number of small fibers in the untreated CAPN3-KO control muscle is of STO histochemical fiber type (Fig. 3e, f). Collectively, these findings show that CAPN3 replacement via gene therapy in the CAPN3-KO muscle rescued defective regeneration, evidenced with toward normalization of fiber size and a decrease in the number of STO fiber population.

**Table 2:** Tibialis anterior muscle fiber size in CAPN3-KO mice following gene therapy

CAPN3-KO	Untreated-TA		AAV.CAPN3.wt-treated TA	
	Number per square millimeter	Diameter (μm)	Number per square millimeter	Diameter (μm)
STO	355	32.72 ± 0.4	223	39.81 ± 0.6^****^
FTG/O	116	44.26 ± 0.9	99	50.40 ± 1.2^****^
All fibers	471	35.55 ± 0.4	322	43.08 ± 0.6^****^

^****^ *P* < 0.0001 compared to untreated parameter

### Impaired regeneration in CAPN3 null muscle is associated with microRNA dysregulation and TGF-β upregulation

**Fig. 4:**
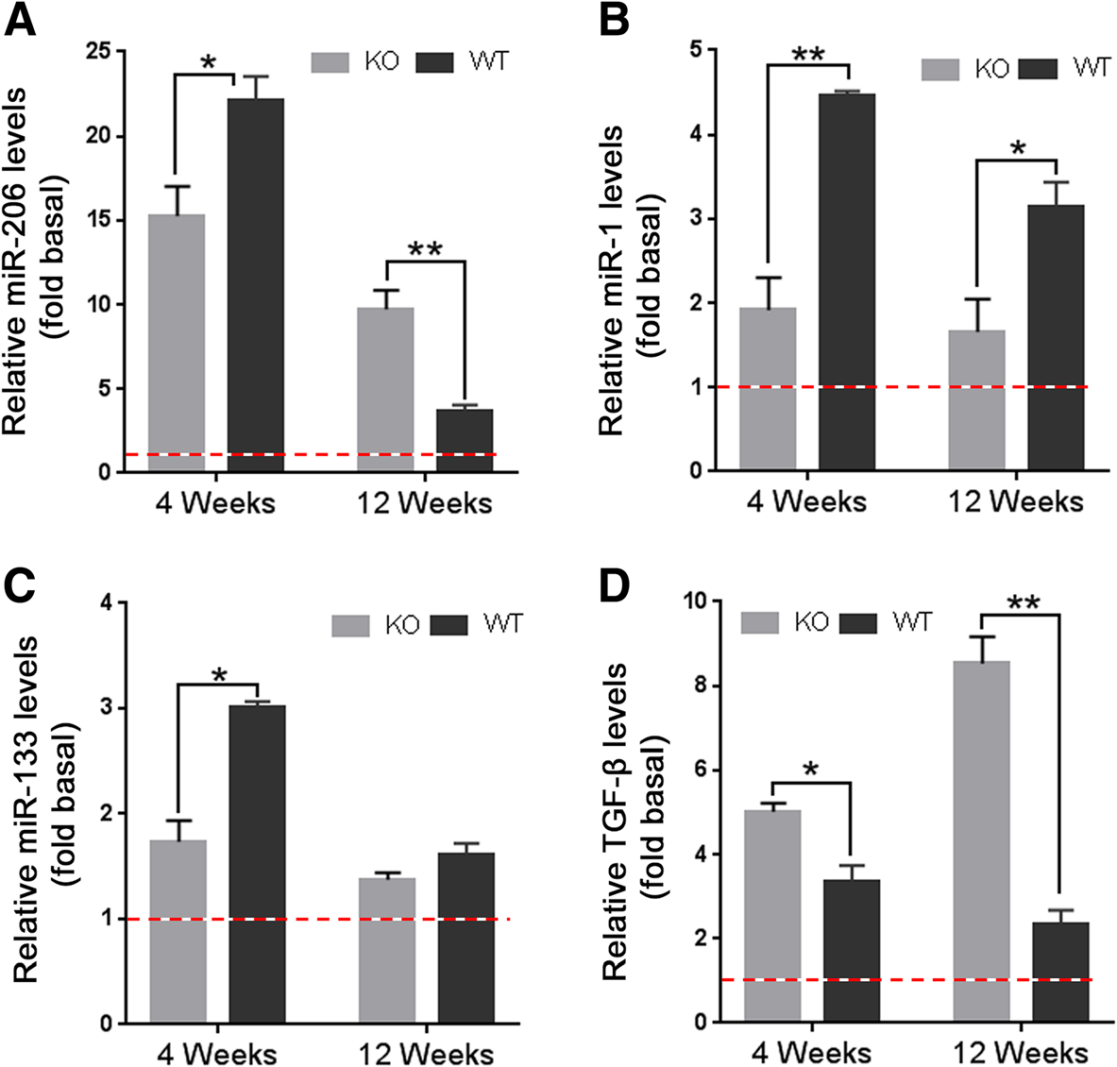
Expression levels of muscle-specific miRNAs and TGF-β in the regenerating gastrocnemius muscles from CAPN3-KO (KO) and wild type (WT) at 4 and 12 weeks post CTX injection. mirR-206 (**a**), miR-1(**b**), miR-133a (**c**), and TGF-β (**d**) levels in the regenerating CAPN3-KO and WT are relative to their baseline levels (dashed line), obtained from the uninjected muscles of 4 weeks post injection cohorts in each group. Expression levels were assessed by qPCR. For miRNAs, internal control was U6 snRNA, and for TGF-β, it was GAPDH. *P < 0.05, **P < 0.01 by two-tailed *t* test. Error bars represent ± SEM; *n* = 3–4 in WT and *n* = 4–5 in KO

Muscle-enriched microRNAs are involved in the regulation of myogenic commitment and skeletal muscle formation, and they may also have significant roles in pathological settings [28]. miR-206 plays a critical role as it acts to promote SC differentiation and fusion into muscle fibers, involved in muscle regeneration [29, 30]. Previously, in muscles from LGMD2A patients, we found Pax7-positive SCs were highest in specimens from older patients with longer disease duration, correlating with downregulation of miR-1, miR-133a, and miR-206 [12]. In the current study, we first assessed the expression levels of these microRNAs following repeated cycles of necrosis/regeneration events in CAPN3-KO and WT muscles. We found no statistical differences between myomiR expression levels of uninjected contralateral muscles from WT and CAPN3-KO at 4 and 12 week time points (Additional file 2: Figure S2 A and B). Therefore, data from the regenerating muscles were normalized to the levels obtained from the uninjected muscles of 4 weeks post injection cohorts as baseline in each group. At 4 weeks post injection, miR-206 transcript levels in the CTX-treated WT muscle were 22-fold higher than the baseline levels which came down to 3. 6-fold dramatically at 12 weeks post last CTX injection (Fig. 4a). In the CAPN3-KO regenerating muscle, the miR-206 transcript levels at 4 weeks were 15-fold of baseline and by 12 weeks came down to 10-fold of baseline levels. This suggests that miR-206 transcripts are significantly lower than WT during the earlier time point and followed an attenuated course, coming down with a slower rate than the WT as seen at 12 weeks post injection. The change in expression levels of miR-1 (Fig. 4b) and miR-133a (Fig. 4c) were also lower than that in the WT counterparts at both time points followed a similar pattern of slower decline.

TGF-β is a potent inhibitor of myogenic differentiation that acts by regulating the expression of miR-206 through regulation of HDAC4 [31]. In addition, TGF-β activity and signaling are involved in broad spectrum of developmental disorders and major pathologies including cancer, fibrosis, and autoimmune diseases. At 4 weeks post injection, TGF-β transcript levels in the regenerating CAPN3-KO muscle was fivefold higher than the baseline levels which was significantly higher than the regenerating WT counterpart (Fig. 4d). At 12 weeks post injection, we observed a decline in the TGF-β transcription levels in the regenerating WT muscles (from threefold to twofold of baseline). In contrast, TGF-β expression levels in the regenerating CAPN3-KO muscles continued to increase, reaching up to 8.5-fold of baseline level and correlating with increased connective tissue content (Fig. 4d). It is of note that reference genes, GAPDH and U6 snRNA, were expressed uniformly across the samples in all real-time PCR experiments (Additional file 3: Figure S3 A and B).

### Akt/mTORC1 activity is perturbed in CAPN3 null muscle

**Fig. 5:**
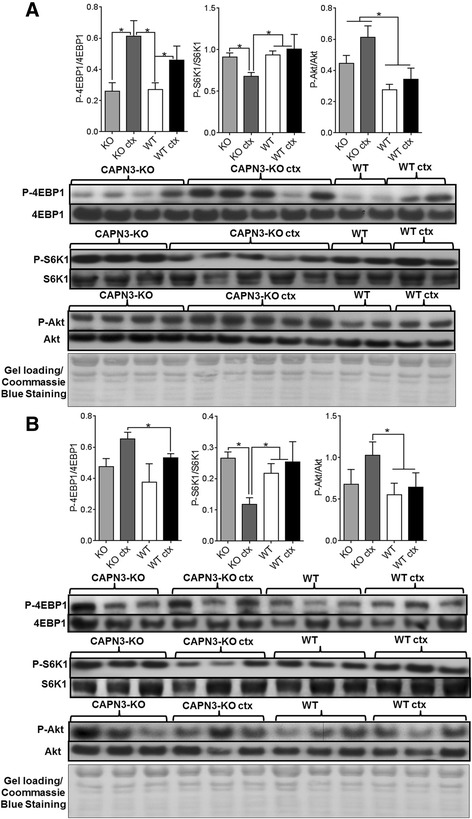
Representative western blot images and analysis of Akt/mTOR signaling in CAPN3-KO (KO) and wild type (WT) gastrocnemius muscles. Analysis of P-Akt (Thr^308^) and mTOR targets, P-4EBP1 (Thr^37/46^), P-S6 K (Thr^389^) at 4 weeks (**a**) and 12 weeks (**b**) post CTX injection. The results show expression levels of the phosphorylated form of proteins normalized to non-phosphorylated forms. *P < 0.05, two-tailed *t* test. Error bars represent ± SEM; *n* = 3–4 in WT and *n* = 4–5 in KO. Coomassie Blue stained membranes were shown as representative loading controls

The mammalian target of rapamycin, mTOR, is a central regulator of cellular growth and metabolism. mTOR signaling senses and integrates a variety of environmental cues to regulate muscle mass and coordinates protein and lipid synthesis, oxidative metabolism, and glucose homeostasis, activated in response to growth factors via the PI3K/Akt pathway [16, 32]. The activity of mTORC1 was assessed by phosphorylation levels of its substrates, the downstream eukaryotic translation initiation factor 4E (eIF4E)-binding protein1(4E-BP1) and ribosomal proteinS6 kinase (S6K). Via 4E-BPs, mTORC1 regulates synthesis of nucleus-encoded mitochondrial proteins, controls mitochondrial activity and biogenesis, and therefore, coordinates energy consumption and production [15]. At 4 weeks post CTX injection, in both CAPN3-KO and the WT, the phosphorylated 4E-BP1 levels were significantly increased in the regenerating muscles compared to uninjured counterparts and this increase was more prominent in the regenerating CAPN3-KO. At 12 weeks post CTX injection, there was no difference between the phosphorylated 4E-BP1 levels of regenerating and WT muscles; however, 4E-BP1 remained highly phosphorylated in the regenerating mutant muscle compared to WT counterpart (Fig. 5b). In contrast, we observed an opposite pattern for S6K1 phosphorylation in the mutant muscles. At 4-week time point, the phosphorylated S6K1 levels showed some increase only in the regenerating WT muscles without reaching statistical significance while in the regenerating CAPN3-KO muscle the levels were significantly lower than the uninjured KO as well as regenerating WT counterparts (Fig. 5a). At 12 weeks post CTX injection, there was no difference between the phosphorylated S6K1 levels of regenerating and uninjured WT muscles while in the CAPN3-KO, the phosphorylation levels of S6K1 remained significantly low in the regenerating CAPN3-KO muscles compared to uninjured KO muscles and the WT counterparts. (Fig. 5b).

As the next step, we assessed the Akt activity in the regenerating and non-regenerating CAPN3-KO and WT muscles and found increased phosphorylated Akt (on Thr^308^) levels only in the regenerating CAP3-KO muscles compared to uninjured counterparts at 4 weeks post injection (Fig. 5a, b). At both time points, Akt activation in the regenerating CAPN3-KO muscles were significantly higher compared to WT counterparts. Collectively, these results show that Akt/mTORC1 signaling pathway is perturbed in the regenerating CAPN3-KO muscle.

### Attenuated mitochondria biogenesis and persistent activation of AMPK in regenerating CAPN3 null muscle

**Fig. 6:**
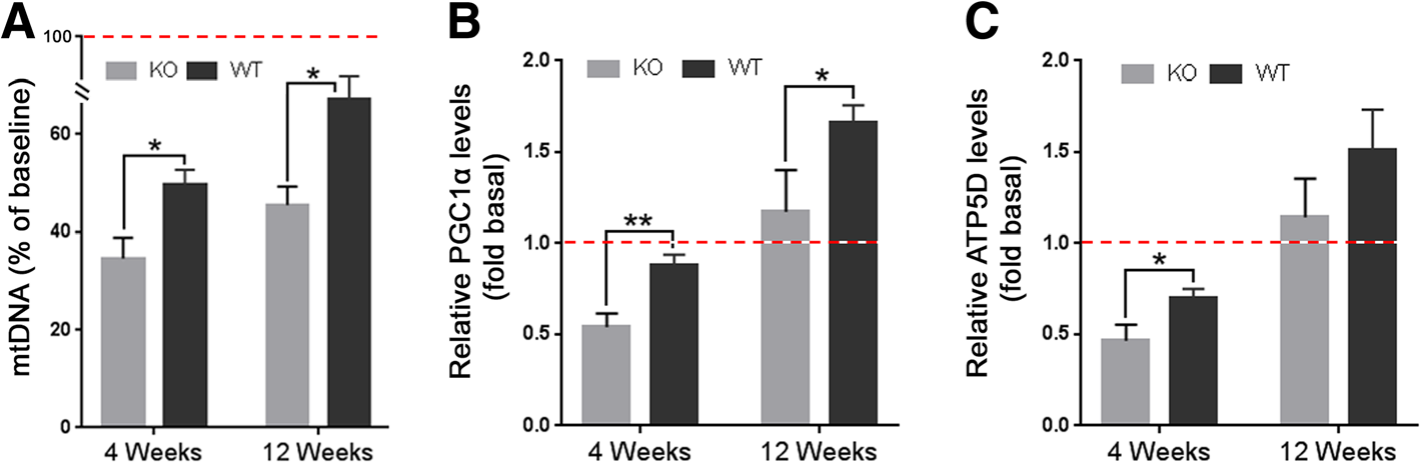
Analysis of mitochondrial content and the expression of mitochondrial biogenesis markers in the regenerating gastrocnemius muscles from CAPN3-KO (KO) and wild type (WT) at 4 and 12 weeks post CTX injection. Mitochondria content (mtDNA/nDNA) of regenerating muscles (**a**) are expressed as percent of their baseline levels (dashed line), obtained from the uninjected muscles of 4 weeks post injection cohorts in each group. The expression levels of PGC1α (**b**) and ATP5D (**c**) in the regenerating muscles from CAPN3-KO and WT are relative to their baseline levels (dashed lines) in the uninjected contralateral muscles of 4 weeks post injection cohorts in each group. Expression levels were determined by qPCR. PGC1α and ATP5D expressions were normalized to GAPDH expression. *P < 0.05, **P < 0.01 by two-tailed *t* test. Error bars represent ± SEM; *n* = 3–4 in WT and *n* = 4–5 in KO

Skeletal muscle fibers differentiate into specific fiber types with distinct metabolic properties determined by their reliance on oxidative phosphorylation. Fatigue resistant oxidative fibers have high mitochondria content; despite their high capacity for protein synthesis, these fibers remain relatively small [33]. In the regenerating CAPN3-KO muscles, we found excessive number of small STO fibers, more prominent at 12 weeks post CTX injection. Skeletal muscle-specific activation of 4EBP1 and switch to STO phenotype was shown previously [34]. To assess the status of mitochondria biogenesis in the setting of perturbed Akt/mTORC1 activity, we first determined the mtDNA content in the baseline and regenerating CAPN3-KO muscles and WT counterparts. During regeneration, we found that the mtDNA contents of CAPN3-KO and WT muscles were lower than those of the uninjured levels. At 4 weeks post CTX injection, in the WT regenerating muscle, mtDNA content was 49% of uninjured muscle levels which increased to 67% at 12 weeks, corresponding to an 18% increase during the 8-week interval (Fig. 6a). In contrast, mtDNA content of the regenerating CAPN3-KO muscle at 4 weeks post injection was much lower, 34% of the uninjected muscle, which showed a meager increase, then went up to 45% by 12-week time point.

We then examined the expression levels of PGC-1α, a key regulator of mitochondrial biogenesis and a marker of mitochondria function, ATP synthase subunit delta (ATP5D) using real-time qPCR [35–37], and found alterations corresponding to the changes seen in mtDNA content. At 4 weeks post CTX injection, the WT regenerating muscles showed lower than baseline expression levels of PGC-1α and ATP5D, which went up a 1.7- and 1.5-fold increase compared to baseline respectively, at 12-week time point (Fig. 6b, c). In the regenerating CAPN3-KO muscle at 4-week time point, PGC-1α and ATP5D transcripts normalized to baseline were significantly lower compared to WT counterparts. At 12 weeks post injection, both PGC-1α and ATP5D transcripts reached to baseline levels although remained significantly lower than the WT counterparts (Fig. 6b, c). Collectively, these results indicate that in the regenerating CAPN3 null muscle, the increases in mtDNA content, mitochondria biogenesis, and ATP production are significantly attenuated compared to the WT which could result in a significant energy and protein synthesis deficit.

#### Activation of AMPK

As the next step, we focused on the AMPK signaling which regulates energy hemostasis in the cell, induced by cellular stress, shortage of ATP (increased AMP/ATP ratio), and increased free radicals [24, 38]. AMPK has critical roles in coordinating cell growth and autophagy, and it also has recently been connected to regulation of many other physiological events including mitochondrial function such as fatty acid oxidation, and mitochondrial biogenesis and in response to a rise in intracellular Ca^2+^ [39]. AMPK acts as a main sensor for ATP deficit inhibiting high ATP consuming mTORC1 pathway by directly phosphorylating the mTORC1 binding regulatory domain, raptor on Ser^792^, resulting in decreased protein synthesis [40]. Activation of AMPK occurs when it is phosphorylated on threonine 172 (Thr^172^) within its catalytic subunit [41] and active AMPK can also trigger autophagy through a mechanism of directly phosphorylating autophagy-initiating kinase Ulk1 in response to low nutrient availability and stress [42].

**Fig. 7:**
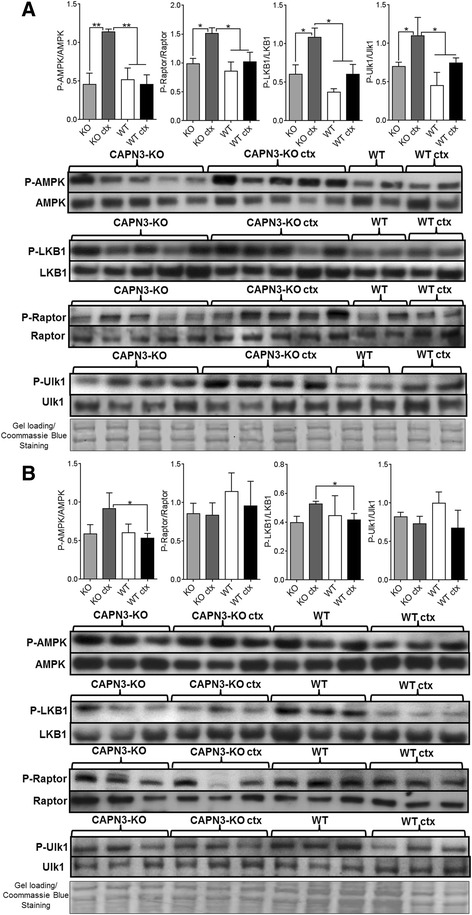
Representative western blot images and analysis of AMPK signaling in CAPN3-KO (KO) and wild type (WT) gastrocnemius muscles. Expression of P-AMPK (Thr^172^), P-LKB1 (Ser^428^), P-Raptor (Ser^792^), and P-Ulk1 (Ser^555^) at 4 weeks (**a**) and 12 weeks (**b**) post CTX injection. The results show expression levels of the phosphorylated form of proteins normalized to non-phosphorylated forms. *P < 0.05, **P < 0.01 two-tailed *t* test. Error bars represent ± SEM; *n* = 3–4 in WT and *n* = 4–5 in KO. Coomassie Blue stained membranes were shown as representative loading controls

We first showed that at 4 weeks post CTX injection, the phosphorylated AMPK (on Thr^172^) levels were significantly higher in the regenerating CAPN3-KO muscles compared to contralateral uninjured KO and WT (uninjured or regenerating) values (Fig. 7a). At 12 weeks, the AMPK activation in the regenerating CAPN3-KO muscles remained high compared to regenerating WT muscle (Fig. 7b). This was associated with higher phosphorylation of raptor on Ser^792^ in the regenerating CAPN3-KO muscle only at 4 weeks post injection time point; no change in the levels of phosphorylated raptor was seen between samples at 12 week (Fig. 7a, b). We then examined the phosphorylation status of two upstream activators of AMPK, liver kinase B1 (LKB1) and Ca^2+^/calmodulin-dependent protein kinase kinase 2 (CaMKK2) [43]. LKB1 mediates activation by the AMP-dependent pathway, while CaMKK2 mediates the Ca^2+^-dependent pathway [44, 45]. In parallel to the activated AMPK levels, phosphorylated LKB1 levels were significantly high in the regenerating CAPN3-KO muscles compared to uninjured KO and the regenerating WT muscles at 4 weeks post injection and remained high compared to the regenerating WT at 12-week time point (Fig. 7a, b). On the other hand, the CaMKK2 levels showed no change between the regenerating and uninjured muscles from both CAPN3-KO and WT at both time points (not shown). Based on these findings, we conclude that LKB1 is the predominant activator of AMPK in the regenerating CAPN3-KO muscle. In addition, phosphorylated Ulk1 level was significantly high in the regenerating CAPN3-KO muscle compared to baseline KO and WT counterparts at 4 weeks post CTX injection indicating active microautophagy as sign of increased catabolic activity (Fig. 7a). At 12-week time point, Ulk1 phosphorylation levels showed no statistical difference between groups (Fig. 7b).

## Discussion

As a cellular process, regeneration promotes high levels of protein synthesis, and mRNA translation alone is considered one of the most energy-consuming processes [15, 16]. It has been well established that muscle fiber size and oxidative capacity are determined by the balance between myofibrillary protein synthesis, mitochondrial biosynthesis, and degradation. Fatigue-resistant oxidative fibers with high mitochondria content, despite their high capacity for protein synthesis, remain relatively small, and we believe this “fiber type-fiber size paradox,” recognized previously [33], has direct relevance to our studies. High oxidative fibers show higher densities of myonuclei and mitochondria, and mitochondrial biogenesis requires both mitochondrial and nuclear DNA, their capacity for mitochondrial protein synthesis is higher compared to the low oxidative fibers. Available data suggests that the low oxidative muscle fibers have a larger potential to phosphorylate S6K1 compared to high oxidative fibers, in relation to increases in translation initiation and subsequent gain in muscle mass [46–48]. Competing interactions between mTORC1 and AMPK pathways are also recognized as determinants of fiber size. AMPK stimulates oxidative metabolism and slow-type gene expression through PGC-1α, while AMPK simultaneously attenuates protein synthesis by inhibiting mTORC1. Akt/mTORC1 and AMPK was noted to act as a “switch” that controls the synthesis of myofibrillar and metabolic proteins [49, 50], implying that the cellular energy status may be crucial in mediating either a low oxidative phenotype and a large size or a high oxidative phenotype and a small size [33].

In the regenerating WT muscle, we observed activation of 4E-BP1 only at the 4-weeks post injection time point. Increases in the phosphorylated levels of 4E-BP1 at 12 weeks and S6K1 and Akt at both 4 and 12 weeks post injection were not significant compared to uninjured WT muscle. There was also no evidence of AMPK activation in the regenerating WT muscle at these two time points that we studied. Collectively, these results suggest that in the WT regenerating muscle after 4 weeks post CTX injection, activities in the Akt/mTOR and AMPK signaling pathways have almost reached to a state of homeostasis.

In the regenerating CAPN3-KO, we found several abnormalities as evidence for competing interactions between Akt/mTORC1 and AMPK pathways, leading to impaired radial growth dynamics. Activation of 4E-BP1 appeared uncoupled from activation of S6K1, as we found significantly low levels of S6K1 in the regenerating mutant muscles. This finding paralleled with significantly smaller fiber size of all fiber types in the CAPN3-KO compared to WT at 12 weeks post injection time point. Furthermore, during early time point, 4 weeks after last CTX injection, we showed that AMPK, the main stress sensor for cellular ATP shortage along with its upstream kinase LKB1 in the cell, was highly activated in the regenerating CAPN3-KO muscles and provided evidence that the activated AMPK suppressed mTORC1 activity by directly phosphorylating the mTORC1-binding regulatory domain raptor, on Ser^792^. Other potential mechanisms of AMPK-mediated inhibition of mTORC1, such as phosphorylation of TSC-2 and eEF-2, remain to be investigated.

AMPK activation was associated with highly phosphorylated Ulk1 levels in the regenerating CAPN3-KO muscle compared to the uninjured KO as well as the regenerating and uninjured WT levels at 4 weeks post CTX injection indicating an increased catabolic activity to control fiber size. Interestingly, Raptor and Ulk1 activation in the regenerating CAPN3-KO was observed only at 4 weeks post injection; an increase in fiber size of all fiber types occurred at 12 weeks compared to 4 weeks. This might suggest that at 12 weeks, mTOR inhibition by AMPK has declined resulting in a delayed transition of muscle fibers from highly oxidative catabolic state to anabolic state or these opposing metabolic pathways have reached to an equilibrium.

Dysregulation of Ca^2+^ metabolism was implicated to play an important role in the pathogenesis of LGMD2A [51], and a recent study reported Ca^2+^/calmodulin-dependent protein kinase 2 (CaMK2) is downregulated in CAPN3-KO muscle after exercise, giving rise to insufficient upregulation of PGC-1α through p38 mitogen-activated protein kinase (MAPK) signaling [52]. In the same study, exercise-induced changes in AMPK levels were not found between WT and CAPN3-KO muscles, although increased oxidative stress along with findings compatible with mitochondria dysfunction and low ATP production in 1-year-old CAPN3-KO muscles were described by the same group as secondary effects of the absence of CAPN3 [53]. In our studies, the regenerating CAPN3-KO muscles showed a failure to increase mtDNA content, PGC-1α, and ATP5D transcripts compared to the regenerating WT counterparts. Yet, the number of small STO fibers in the regenerating CAPN3-KO muscles was much higher than the WT counterpart. One plausible explanation for this “mismatch” is that individual premature STO fibers continue to display high mitochondria density relative to their small size. Mitochondria biogenesis is under the control of several pathways. p38/MAPK and CaMK2 signaling pathways are well-characterized upstream modulators of PGC-1a expression in skeletal muscle [54, 55]. More recently, skeletal muscle-specific persistent activation of 4E-BP1 and switch to STO phenotype was shown via mitochondria biogenesis, presumably through increased translation of PGC-1α [34]. Our findings in the regenerating WT muscles are in fact displaying this relationship between the activated 4E-BP1 and switch to small diameter STO fiber phenotype. In response to 4E-BP1 activation, the regenerating WT muscle produced close to uninjected baseline levels of PGC-1α expression at 4 weeks and a 1.7-fold increase of baseline at 12 weeks post CTX injection. Considering the mechanism of CTX-injury model, which damages myofiber mitochondria [56], an efficient mitochondrial biogenesis seems taking place in the WT muscle during recovery. In contrast, highly activated 4E-BP1 in the regenerating CAPN3-KO muscle is incapable of inducing mitochondrial biogenesis with same efficiency as in the WT. In addition, mTORC1 was not capable of activating S6K, whereas in the regenerating WT muscle, S6K activation have reached or exceeded uninjured WT muscle levels at 4 weeks post CTX injection. Taken together, this differential activation of 4E-BP1 and S6K by mTORC1 is likely to have caused significantly smaller oxidative fibers containing high mitochondria density for their size, while the overall mitochondria content is less than WT.

Our studies showed significantly increased TGF-β transcription levels in the regenerating CAPN3-KO muscles associated with downregulation of myomiRs and increased fibrosis. Interestingly, the pattern of alterations in these myomiRs was similar to our previous findings [12]. In muscle biopsied from LGMD2A patients, Pax7-positive SCs were highest in the fibrotic group and correlated with microRNA dysregulation as downregulation of miR-1, miR-133a, and miR-206 [12]. We found miR-206 modestly upregulated (1.6- to twofold change) in the inflammatory and lobulated (biopsies with excessive number of lobulated fibers) groups, but downregulated below control levels in the fibrotic group representing an advanced stage of the calpainopathy. It should be noted that the level of miR-206 upregulation seen in prior to the very late phase of calpainopathy is modest compared to examples of robust regeneration as seen in WT muscle following CTX-induced injury where miR-206 increases dramatically within regenerating muscle fibers [57]. Following one round of CTX injection into TA muscle, increased miR-206 expression level in WT remained high for several weeks and was still significantly higher than the pre-injection level even 8 weeks after CTX injection [57]. Our current study showed a sevenfold less miR-206 expression in the regenerating CAPN3-KO muscle compared to WT counterpart at 4 weeks post injection time point and the levels declined slower than WT as seen at 12 weeks post injection. Compared to WT, overall, these changes in the expression levels of the microRNAs in CAPN3-KO mice are comparable to those reported in the X-linked canine dystrophy model following CTX injection [57]. Collectively, these findings show similar patterns of microRNA dysregulation in the regenerating CAPN3-KO muscle as we previously observed in the biopsies from LGMD2A patients. Moreover, along with the downregulation of miR-206, the upregulation of TGF-β underlines its important regulatory role in stress signal mediation process, leading to a phenotypic switch, to fibrosis as proposed previously [28].

We believe our studies in CAPN3-KO mice provide insight into the dystrophic process in LGMD2A. In a previous study, we illustrated the continuum of the dystrophic process from prominent inflammation with ongoing necrosis and regeneration to prominent fibrosis, correlating with age and disease duration [12]. Excessive number of small- and medium-size fibers with high oxidative capacity (type 1) and fibers with lobulated appearance were predominating features of the biopsies from patients with longer disease duration. In this current study, we used repeated cycles of CTX injection paradigm to simulate the early stages of dystrophic process and assessed the radial growth process 4 and 12 weeks after last CTX injection. By doing so, we were able to detect a failure in the radial growth phase of regeneration in the CAPN3-KO muscle, particularly of those oxidative types, while the WT muscle, subjected to the same paradigm, continued to grow in size significantly within the period of 8-week interval. This approach in our hands, forcing cells to switch to regeneration mode, has been useful for studying knockout models exhibiting no phenotype or minimal pathology. The utility of CTX-induced repeated cycles of regeneration paradigm has been shown in ANO5-KO mouse, model for LGMD2L, which we found the presence of perturbed regeneration [58]. In another study, attenuated muscle regeneration was found as a key factor in dysferlin-deficient (C57BL/10.SJL-Dysf) mice with three rounds of notexin injection resulting in a striking dystrophic phenotype [59].

Using the mouse model of CTX injury, fiber-type conversion during muscle repair has been shown previously [60, 61]. In our studies, although the radial growth of WT muscle fibers were significantly better, we found neither the fiber size nor the fiber type proportion had returned to baseline at 12 weeks post CTX injection, although a decrease in the STO fibers alone with size increase was present compared to earlier time point. A previous study reported that fiber-type conversion from fast- to slow-type persisted with a higher percentage of small fast- and slow-type myofibers in the regenerated gastrocnemius muscles of mice 8 months after myotoxin injury (induced by copperhead snake venom) [61]. Although there are no comparable studies with CTX injury (induced by *Naja mossambica* mossambica), we predict that full recovery of the original fiber size may not occur, which might be due to a metabolic reprogramming of the small fibers. In that regard, these findings in the WT are reminisce to those changes seen in partially treated acquired autoimmune myopathies where the muscle is subjected to repeated cycles of necrosis/regeneration, leading to fixed-muscle weakness, ultimately.

## Conclusions

In summary, how the absence of CAPN3 triggers an initial event that leads to metabolic reprogramming in the muscle is not entirely understood at this time. Nevertheless, our study for the first time provided evidence that the metabolic adjustment triggered by the absence of CAPN3 in muscle results in an aberrant regeneration, characterized by an increase in the number of small-size STO fibers with “lobulated” appearance, therefore recapitulating the LGMD2A pathologic phenotype. In the regenerating CAPN3-KO muscle, Akt /mTORC1 pathway was uncoupled from protein synthesis due to direct inhibition of mTORC1 through LKB1/AMPK pathway, known to be triggered by energy shortage. In addition, our in vitro studies showed that the absence of CAPN3-related metabolic remodeling is directly responsible for this aberrant regeneration, causing fusion defective myoblasts and myotubes. Since the final stage and further maturation of myotubes/myofibers is regulated in a mTOR-kinase-dependent manner [62], this early defect is likely to be important in the course of regeneration. Furthermore, we have corrected this growth failure in the CAPN3-KO mice by replacing the missing protein via intramuscular injection of an AAV vector carrying the *CAPN3* gene. These experiments will pave the road to preclinical gene therapy protocols in which the *CAPN3* should be expressed under the skeletal muscle-specific tMCK promoter for systemic administration to prevent previously reported cardiac toxicity [63].

We believe that impaired regeneration/radial growth following necrotic events in muscle is a fundamental feature of a dystrophic process as major contributor to fibrosis and disability. Our findings demonstrating the essential role of the activation of AMPK pathway leading to impaired radial growth in CAPN3-KO muscle is an important one with potential implications to other dystrophies and chronic myopathic conditions. It will require further studies to determine whether this pattern of metabolic remodeling leading to attenuated regeneration represents a common pathway in disorders due to markedly disparate gene defects. Understanding common features should allow development of novel therapeutic strategies.

## Additional files

Additional file 1: Figure S1. (A-D) Increased oxidative fibers and impaired radial growth in regenerating CAPN3-KO muscle. Representative images of SDH-stained tissue sections of CAPN3-KO (A, C) and wild type (WT) gastrocnemius muscles (B, D) at 4 and 12 weeks after last CTX injection, respectively. The sections were double stained for type 1 (green) and type 2A (red) with MHC antibodies. Increased number of small type I (slow twitch oxidative) and type IIA (fast twitch oxidative) fibers are noted in the CAPN3-KO muscle compared to WT. A few hybrid fibers (yellow) are seen in A. Scale bar = 30 μm. (TIFF 12944 kb)

Additional file 2: Figure S2. A and B. Comparison of baselines in uninjected KO and WT muscles at 4 weeks (A) and 12 week time points (B). Relative quantity of all markers were assessed by qPCR. For miRNAs, internal control was U6 snRNA. For TGF-β, PGC1α and ATP5D it was GAPDH. mtDNA was normalized to nDNA values in each group Error bars represent ± SEM; *n* = 3–4 in WT and *n* = 4–5 in KO. (TIFF 1723 kb)

Additional file 3: Figure S3. A and B. GAPDH and U6 snRNA were expressed uniformly across the samples in all real-time PCR experiments at 4 weeks (A) and 12 weeks (B) time point analyses. Ct value data is presented as box-whisker plots; the boxes represent the lower and upper quartiles with lines in between representing medians; whiskers represent the data from KO (n = 4–5) and WT (n = 3–4) animals run in duplicates (C and D). (TIFF 1542 kb)

## Abbreviations

AAV: Adeno-associated virus
ATP5D: ATP synthase subunit delta
CAPN3-KO: Calpain-3 knockout
CTX: Cardiotoxin
FTG: Fast twitch glycolytic
FTO: Fast twitch oxidative
LGMD2A: Limb-girdle muscular dystrophy type 2A
LKB1: Liver kinase B1
MCK: Muscle creatine kinase
miRNA: MicroRNA
mTOR: Mechanistic target of rapamycin
P-4EBP1: Phosphorylated eukaryotic translation initiation factor 4E–binding protein 1
P-Akt: Phosphorylated protein kinase B
P-AMPK: Phospohrylted AMP-activated protein kinase
PGC1α: Peroxisome proliferator-activated receptor gamma coactivator 1-alpha
P-S6 K1: Ribosomal protein S6 kinase beta-1
STO: Slow twitch oxidative
TGF-β: Transforming growth factor beta
WT: Wild type
