# Supplementary figures and images for: Impaired regeneration in calpain-3 null muscle is associated with perturbations in mTORC1 signaling and defective mitochondrial biogenesis
Source: Skelet Muscle. 2017 Dec 14;7:27. doi: 10.1186/s13395-017-0146-6 (PMC5731057; doi:10.1186/s13395-017-0146-6)

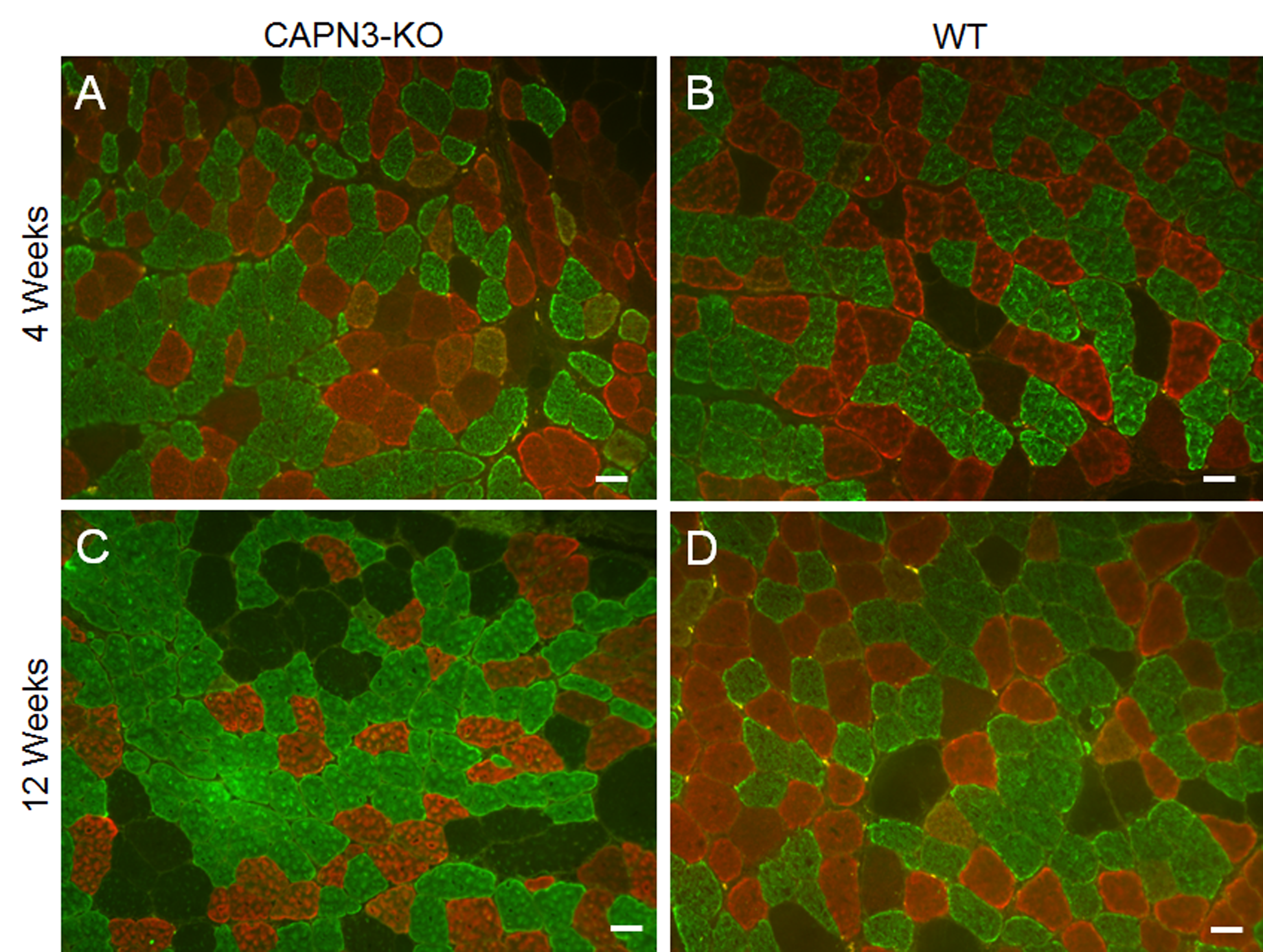

Supplement: Supplementary file 1 — (A-D) Increased oxidative fibers and impaired radial growth in regenerating CAPN3-KO muscle. Representative images of SDH-stained tissue sections of CAPN3-KO (A, C) and wild type (WT) gastrocnemius muscles (B, D) at 4 and 12 weeks after last CTX injection, respectively. The sections were double stained for type 1 (green) and type 2A (red) with MHC antibodies. Increased number of small type I (slow twitch oxidative) and type IIA (fast twitch oxidative) fibers are noted in the CAPN3-KO muscle compared to WT. A few hybrid fibers (yellow) are seen in A. Scale bar = 30 μm. (TIFF 12944 kb) [file 13395_2017_146_MOESM1_ESM.tif]

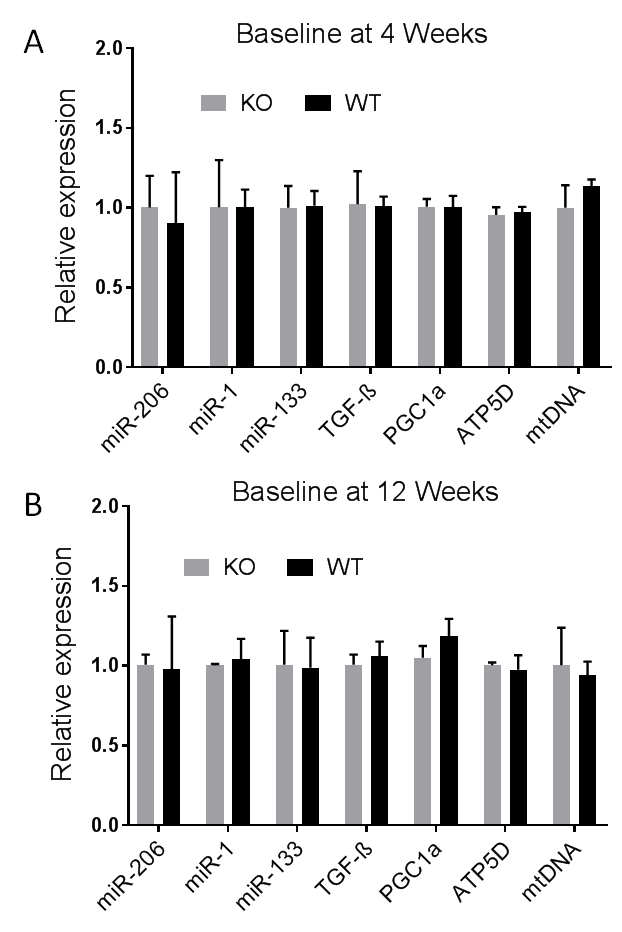

Supplement: Supplementary file 2 — A and B. Comparison of baselines in uninjected KO and WT muscles at 4 weeks (A) and 12 week time points (B). Relative quantity of all markers were assessed by qPCR. For miRNAs, internal control was U6 snRNA. For TGF-β, PGC1α and ATP5D it was GAPDH. mtDNA was normalized to nDNA values in each group Error bars represent ± SEM; n = 3–4 in WT and n = 4–5 in KO. (TIFF 1723 kb) [file 13395_2017_146_MOESM2_ESM.tif]

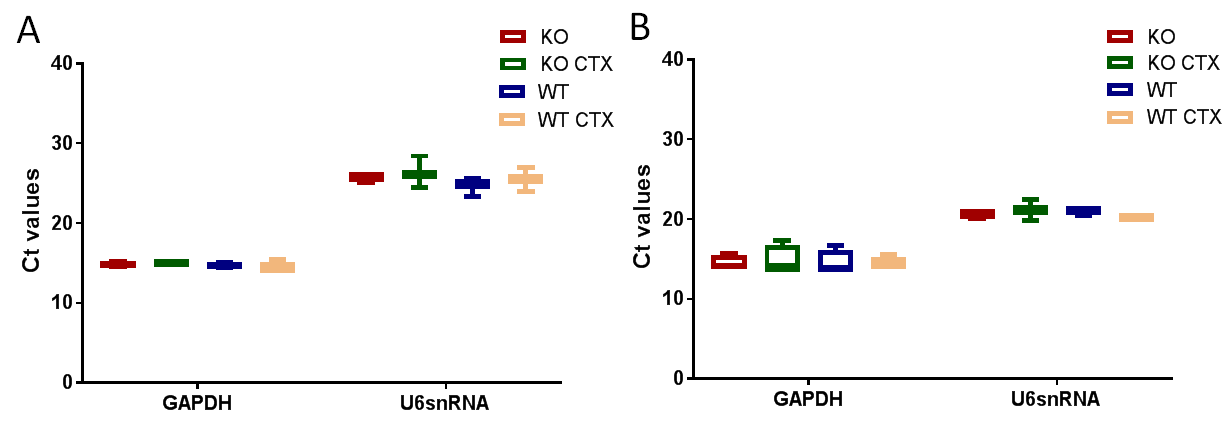

Supplement: Supplementary file 3 — A and B. GAPDH and U6 snRNA were expressed uniformly across the samples in all real-time PCR experiments at 4 weeks (A) and 12 weeks (B) time point analyses. Ct value data is presented as box-whisker plots; the boxes represent the lower and upper quartiles with lines in between representing medians; whiskers represent the data from KO (n = 4–5) and WT (n = 3–4) animals run in duplicates (C and D). (TIFF 1542 kb) [file 13395_2017_146_MOESM3_ESM.tif]
